# The impact of surfactant protein-A on ozone-induced changes in the mouse bronchoalveolar lavage proteome

**DOI:** 10.1186/1477-5956-7-12

**Published:** 2009-03-26

**Authors:** Rizwanul Haque, Todd M Umstead, Willard M Freeman, Joanna Floros, David S Phelps

**Affiliations:** 1 Penn State Center for Host defense, Inflammation, and Lung Disease (CHILD) Research and the Department of Pediatrics, Penn State College of Medicine, Hershey, PA, USA; 2 The Department of Pharmacology, Penn State College of Medicine, Hershey, PA, USA; 3 The Department of Obstetrics and Gynecology, Penn State College of Medicine, Hershey, PA, USA

## Abstract

**Background:**

Ozone is a major component of air pollution. Exposure to this powerful oxidizing agent can cause or exacerbate many lung conditions, especially those involving innate immunity. Surfactant protein-A (SP-A) plays many roles in innate immunity by participating directly in host defense as it exerts opsonin function, or indirectly via its ability to regulate alveolar macrophages and other innate immune cells. The mechanism(s) responsible for ozone-induced pathophysiology, while likely related to oxidative stress, are not well understood.

**Methods:**

We employed 2-dimensional difference gel electrophoresis (2D-DIGE), a discovery proteomics approach, coupled with MALDI-ToF/ToF to compare the bronchoalveolar lavage (BAL) proteomes in wild type (WT) and SP-A knockout (KO) mice and to assess the impact of ozone or filtered air on the expression of BAL proteins. Using the PANTHER database and the published literature most identified proteins were placed into three functional groups.

**Results:**

We identified 66 proteins and focused our analysis on these proteins. Many of them fell into three categories: defense and immunity; redox regulation; and protein metabolism, modification and chaperones. In response to the oxidative stress of acute ozone exposure (2 ppm; 3 hours) there were many significant changes in levels of expression of proteins in these groups. Most of the proteins in the redox group were decreased, the proteins involved in protein metabolism increased, and roughly equal numbers of increases and decreases were seen in the defense and immunity group. Responses between WT and KO mice were similar in many respects. However, the percent change was consistently greater in the KO mice and there were more changes that achieved statistical significance in the KO mice, with levels of expression in filtered air-exposed KO mice being closer to ozone-exposed WT mice than to filtered air-exposed WT mice.

**Conclusion:**

We postulate that SP-A plays a role in reactive oxidant scavenging in WT mice and that its absence in the KO mice in the presence or absence of ozone exposure results in more pronounced, and presumably chronic, oxidative stress.

## Introduction

Ozone is an air pollutant that is known to have a variety of deleterious effects on the human lung [1-6]. These include inflammation, increased airway reactivity, and an increased susceptibility to infection. Ozone exposure has been reported to disrupt epithelial integrity, impair effective phagocytosis, and compromise mucociliary clearance [1]. However, other studies where increased epithelial permeability and changes in ventilation are not observed indicate that these effects may be highly ozone dose-dependent [5]. Ozone effects are more pronounced in asthmatics [4], especially children [3]. Interestingly, ozone-induced inflammation, as measured by neutrophil influx and IL-8 levels, differs between normal subjects and asthmatics, but does not correlate with pulmonary function changes [2]. Differences in the response to ozone among individuals having polymorphisms in genes related to oxidative stress implicate oxidative stress in these processes and provide a basis for varying susceptibility to ozone-induced symptoms [7].

Mechanisms involved in ozone-induced lung damage have been investigated in animal models [8-14]. In general, experimental animals require significantly higher doses of O_3 _exposure than humans [15] to reach comparable amounts of O_3 _concentration in the distal lung. Measurement of various parameters in bronchoalveolar lavage (BAL) revealed that resting rodents exposed to high O_3 _doses (2 ppm) were either comparable (polymorphonuclear leukocytes (PMNs), protein) or lower (macrophages) than the exercising human exposed to considerably lower O_3 _exposures (0.44 ppm). Therefore, it is necessary that rodents be exposed to high O_3 _concentrations to better enable extrapolation of findings from animal studies to human. Our laboratory has demonstrated ozone-dependent changes in mice in epithelial permeability, inflammatory mediators, and susceptibility to pneumonia [8,9,16]. The changes in epithelial permeability have been attributed to TLR-4-mediated changes in iNOS activity [12]. A role for oxidative stress in ozone-induced pathophysiology has been postulated based on increases in F_2_-isoprostane [13], a lipid peroxidation product, as well as reductions in inflammatory mediators and allergen sensitivity by antioxidant treatment [10]. The involvement of oxidative stress is further supported by studies in which genetic polymorphisms influence the response to ozone [17]. Although the pathophysiology of ozone-induced lung damage is incompletely understood, these mechanistic and genetic association studies provide a strong rationale for oxidative stress [7] playing a key role in the response to ozone exposure.

Host defense function is one of the many processes that can be disrupted by oxidative stress. Ozone has been implicated in increasing susceptibility to infection in humans [18,19] and in a number of animal studies (reviewed in [1]), as have other sources of oxidative stress such as sublethal hyperoxia [20]. The basis for these effects is not known, but may relate to the oxidative modification of molecules involved in innate immune processes by reactive oxidant species, lipid peroxidation products, or other molecules generated by oxidative stress. Oxidation of protein molecules can interfere with their function and alter their metabolism by either promoting their degradation or causing the formation of protein aggregates that are not readily degraded [21,22].

Surfactant protein-A (SP-A), a major component of BAL, is an example of an innate immune protein whose function is disrupted by oxidation. SP-A is known to play a variety of roles in innate immune function. These include serving as an opsonin for the recognition of some pathogens [23,24], regulating the production of cell surface antigens and inflammatory mediator expression by some immune cells [25,26], participating in the development of dendritic cells [27], regulating reactive oxidant production [28,29], and others [30]. However, a series of studies from our laboratory has shown that several of these functions are compromised when SP-A is oxidized)[9,31-34]. A number of studies have explored the function of SP-A *in vivo *by subjecting SP-A-/- (SP-A knockout; KO) mice to various infectious or environmental challenges. These include studies of susceptibility to bacterial infection [35,36], susceptibility to viral infection [37,38], oxidant-mediated killing of mycoplasma [39], response to ozone exposure [8,16], and the impact of ozone exposure on susceptibility to pneumonia [16]. These *in vivo *studies have confirmed the diversity of SP-A's influence on innate immune function. Several studies from our laboratory have explored the role of SP-A in vivo in ozone exposure and innate immunity [8,9,16]. We have shown that the response of KO mice to acute ozone exposure, while similar in many respects to that of wild type (WT) mice, has some unique features including the influx of immune cells into the alveolar spaces. KO mice apparently sustain more tissue damage than WT mice, as indicated by BAL lactate dehydrogenase (LDH) levels detectable immediately after a 3 hr ozone exposure. However, at 4 hr after a 3 hr exposure to ozone lower relative numbers of neutrophils were observed in KO mice than WT mice [8], in part explaining the differences in lung mRNA levels for MIP-2, and to a lesser degree for MCP-1, between the two strains. Paradoxically however, no differences were observed in MIP-2 and MCP-1 protein levels between the two strains, underscoring, perhaps, the complexity of the processes involved. We have also shown that ozone exposure increases the susceptibility of mice to infection, at least in part due to the oxidation of SP-A [9], and that KO mice are more susceptible to infection than WT mice [16].

In this study, in order to gain insight into the mechanisms for the studies described above, we employed a discovery proteomic approach to investigate the effects of ozone exposure on the BAL proteome. We also utilized a strain of SP-A KO mice and compared them to WT mice on the same genetic background in order to elucidate the effect of SP-A on these processes. This type of unbiased approach is not dependent upon previously published studies and may be instrumental in generating specific novel hypotheses involving proteins and pathways that may not have been previously implicated in the process being studied. In the case of ozone-induced lung injury each of the studies described above has typically had a very narrow focus, and integrating all of these results into a unified understanding of the pathophysiology of ozone exposure has been difficult [8,40-44].

Preliminary assessments of ozone-induced changes in rat and mouse BAL proteins have used conventional 2-D gel approaches to examine a small group of proteins [45,46]. In one case, differences between an ozone-sensitive strain and an ozone-resistant strain in the response to ozone were explored [46], and in the other, the effects of ozone on 1-nitronaphthalene adduct formation were probed [45]. In the present study we exposed WT and KO mice to ozone or filtered air and studied the resulting changes in the BAL proteome using two-dimensional difference gel electrophoresis (2D-DIGE), a discovery proteomics technique [47-49] for quantitation, coupled with Matrix Assisted Laser Desorption Ionization-Time-of-Flight/Time-of-Flight (MALDI-ToF/ToF) tandem mass spectrometry for identification of proteins. These techniques make it possible to simultaneously analyze hundreds of proteins in biological samples and have helped identify both pathways and additional proteins involved in these pathways in various experimental systems [50-52]. We recently employed a similar approach to examine age-related changes in the rat BAL proteome)[53]. This combination of methods for protein quantification and identification of proteins has proven useful in quantitative comparisons of protein expression and has not been previously applied to a comparison of this kind of SP-A KO mice with WT mice on the same genetic background.

In this study 2D-DIGE and MALDI-ToF/ToF were used to examine the impact of ozone on lung injury in the presence or absence of SP-A, a molecule with an important role in innate immune function. Using the PANTHER database and published literature we assigned many of the proteins identified to three major categories. By comparing the data obtained in WT and KO mice we have put forward a specific and novel hypothesis for the role of SP-A in redox balance and innate immunity in response to ozone-induced oxidative stress.

## Methods

### Animals

The study was conducted with SP-A(+/+) pathogen-free male C57BL/6 mice (wild type; WT) and SP-A-/- (knockout; KO) mice on the C57BL/6 genetic background. WT mice were obtained from Jackson Laboratories (Bar Harbor, ME). Breeder pairs of KO mice were obtained from Dr. Samuel Hawgood at the University of California, San Francisco and propagated in the animal facility at the Penn State College of Medicine. Body weight of the mice ranged from 20–25 g. The animals were bred and maintained under standard environmental conditions and fed rodent chow and tap water ad libitum. The Institutional Animal Care and Use Committee at the Penn State College of Medicine approved this study.

### Experimental Model

A total of 16 five to six week old C57BL/6 WT and KO mice (20–25 g) were divided into four groups with 4 animals per group: 1) WT exposed to filtered air (WTFA); 2) WT exposed to ozone (WTO_3_); 3) KO exposed to filtered air (KOFA); and 4) KO exposed to ozone (KOO_3_). Four mice were put into glass exposure vessels with stainless steel wire mesh lids and then placed in a closed glass exposure chamber. Mice were exposed to either 2 parts/million (ppm) ozone or to filtered air (FA) for 3 hours. Exposures were conducted in parallel at room temperature and 50% humidity as described [8]. The ozone system efficiently delivers ozone concentrations between 0.1 ppm and 10 ppm. Ozone is generated by an electric discharge ozonizer (Model OZ2SS-SS, Ozotech, Yreka, CA) and its concentration is monitored continuously with an ultraviolet ozone analyzer (Model 400A, Advanced Pollution Instrumentation, San Diego, CA, USA). Mice were sacrificed 4 hours after the exposure period ended by anesthetizing them with halothane and exsanguination. The lungs were subjected to BAL with normal saline.

### Total cell and differential cell counts in BAL Fluid

BAL fluid was obtained by instilling saline into the lungs 3 times through a tracheal cannula using a volume equal to 80% of lung vital capacity (for a total of 1.5 ml). Total BAL fluid recovery was approximately 90% of the instilled volume and did not differ significantly between the experimental group and controls. The BAL fluid was centrifuged (150 × g, 10 min, 4°C) and the cell pellet was resuspended in 0.9% sodium chloride. Total cell counts were performed using a hemocytometer and cytocentrifuge preparations were used to obtain differential cell counts. The cell-free BAL supernatant was frozen at -80°C for subsequent proteomic studies.

### Depletion of high abundance serum protein from mouse BAL

Three high abundance serum proteins (albumin, transferrin, IgG) were depleted from mouse BAL by using a Multiple Affinity Removal System (MARS) Spin Cartridge, Ms-3, 0.45 ml resin bed (Agilent Technologies, Inc., Palo Alto, CA) according to the manufacturers recommendations with slight changes. BALs were mixed with an equal volume of lyophilized buffer to avoid further dilution of the BAL and then filtered through a 0.22 micron spin filter. After filtration, 0.2 ml of lavage was run through the MARS cartridge at one time for a total of 6 times for each sample (following the manufacturer's protocol), collecting and pooling the flow-through fractions (F1 and F2) for each, totaling a volume of around 6 ml for each sample. Bound fractions of protein were eluted from the cartridge, totaling a volume of around 12 ml for each sample and saved for further analysis. All of the individual samples were then concentrated by trichloroacetic acid (TCA)/acetone precipitation. In order to assess the completeness of the depletion, separate mouse BAL samples were depleted by passage through the MARS cartridge. The undepleted BAL (cell-free BAL), flow-through fraction (F1 and F2) and bound fraction were each concentrated and desalted by using the supplied Agilent centrifuge concentrators (5000 MWCO, 4 ml). Concentrated samples were resuspended in lysis buffer (30 mM TrisCl, 2 M thiourea, 7 M urea, 4% CHAPS, pH 8.5) for 2-dimensional electrophoresis.

### TCA/Acetone precipitation

One volume of ice cold 100% TCA was added to four volumes of protein sample for each individual pool of flow-through fractions, which were mixed and incubated overnight at 4°C. Following overnight incubation, samples were centrifuged (15,000 × g, 15 min, 4°C) and the protein pellets washed with 250 μl of chilled acetone, centrifuged again, resuspended in a minimum volume of standard cell lysis buffer (30 mM TrisCl, 2 M thiourea, 7 M urea, 4% CHAPS, pH 8.5), and the pH adjusted to a range of 8.0–9.0. Protein determinations were done using the Bio-Rad Protein Assay (Bio-Rad, Hercules, CA) and the concentration of protein was brought to 1 mg/ml for CyDye labeling.

### 2D-DIGE labeling (minimal labeling) and electrophoresis for 2D-DIGE

Information about the 2D-DIGE study is provided in a form that is in concordance with the Minimum Information About a Proteomics Experiment – Gel Electrophoresis (MIAPE-GE) standards [54] currently under development by the Human Proteome Organization Proteomics Standards Initiative (see Additional File 1). Samples from each group were randomly assigned to Cy3 or Cy5 to ensure no dye-based artifacts in quantitation. Aliquots of 12.5 μg of BAL protein from each sample were labeled with Cy3 or Cy5 (200 picomoles). A normalization pool was created by combining equal amounts of protein from every sample (16 samples) and an aliquot of the pool was labeled with Cy2 (200 picomoles). Equal amounts of Cy3-labeled sample, Cy5-labeled sample, and Cy2-labeled pool samples were mixed. The use of a normalization pool is advantageous as this serves as an internal standardization tool for all gels/samples under study, and thus the possibility of erroneous conclusions due to different concentration loads and other related issues is significantly diminished. An equal volume of 2× sample buffer (2 M thiourea, 7 M urea, 2% pH 3–10 nonlinear (NL) IPG buffer, 1.2% DeStreak reagent) was added to all samples including the unlabeled preparative gel sample and then brought up to a volume of 450 μl with rehydration buffer (DeStreak™ Rehydration Solution, 0.5% pH 3–10 NL IPG buffer). Proteins were subjected to isoelectric focusing on 24 cm pH 3–10 NL gradient Immobiline DryStrips (GE Healthcare, Piscataway, NJ) by using an IPGphor II apparatus (GE Healthcare) at 20°C and under mineral oil to prevent evaporation. Proteins were focused by using the following voltages and times: 14 hour at 0 V (passive rehydration); 6 hour at 30 V (active rehydration); 3 hour at 300 V (step and hold); 3 hour at 600 V (gradient); 3 hour at 1000 V (gradient); 3 hour at 8000 V (gradient); 4 hour at 8000 V (step and hold). Each of the strips were equilibrated in equilibration solution-1 (50 mM TrisCl, 6 M urea, 30% glycerol, 2% sodium dodecyl sulphate (SDS), 0.5% dithiothreitol) and equilibration solution-2 (50 mM TrisCl, 6 M urea, 30% glycerol, 2% SDS, 4.5% iodoacetamide) for 15 min respectively. After isoelectric focusing the IEF strips were applied to 10% polyacrylamide gels (26 cm-w × 20 cm-h × 1 mm thick), sealed with 0.5% low melting point agarose containing bromophenol blue in a buffer of 1× Tris/glycine/SDS buffer (25 mM Tris, 192 mM glycine, 0.1% (W/V) SDS, pH 8.3) run overnight at 2 W/gel at 20°C using the EttanDALT system (GE Healthcare) for separation of proteins on the basis of molecular weight. For the preparative picking gel and the gels used to confirm depletion, a single plate for each gel plate sandwich was treated with Bind-Silane solution (80% ethanol, 0.02% glacial acetic acid, 0.001% Bind-Silane) and had reference markers placed on them. After the completion of electrophoresis, the plates that had not been silane-treated were removed from the sandwich and the gels were fixed with 30% methanol, 7.5% glacial acetic acid 2 times for 1 hour.

An aliquot of 125 μg of unlabeled normalization pool was used for the preparative or picking gel to obtain a sample for the identification of the protein spots by MALDI-ToF/ToF. The preparative picking gel and the gels used to confirm depletion were then stained overnight with Sypro Ruby (Invitrogen, Carlsbad, CA) followed by destaining with 10% methanol, 7.5% glacial acetic acid 2 times for 1 hour.

### Gel scanning and image analysis

Information about the acquisition and processing of data from the 2D-DIGE studies are provided in the form recommended for Minimum Information about a Proteomics Experiment – Gel Informatics (MIAPE-GI) currently under development by the Human Proteome Organization Proteomics Standards Initiative http://www.psidev.info/index.php?q=node/83 (see Additional File 2). All two-dimensional gels were imaged on a Typhoon 9410 fluorescent imager (GE Healthcare) at a resolution of 100 μm. Photomultiplier tube voltages were individually set for each of the three colored lasers to ensure maximum, linear signals. The same voltages were used for all the gels. The DIGE Gels were imaged at three different wavelengths (Cy2: 520 nm, Cy3: 580 nm, Cy5: 670 nm) and the Sypro Ruby stained gels were imaged at 100 μm with a separate filter (610 nm).

Gel images were imported into the Progenesis SameSpots v2.0 program (Nonlinear Dynamics, Durham, NC) for analysis. Gel alignment was conducted automatically and then checked manually to ensure correct alignment. A reference gel with minimum distortion and streaks was then selected from the Cy2 gels. Spot detection and spot matching across all the gels was conducted automatically, then spot matching was checked and manually edited to ensure correct matching, merging and splitting of spots.

All the included spots were transported to Progenesis PG240 module of the Progenesis SameSpots v2.0 software. Quantitation of spots was accomplished by comparing the ratio of each Cy3 and Cy5 value to the values obtained from the normalization pool/Cy2 channel present on each gel. Statistical analysis was performed by Student's t-test to confirm the level of significance among various groups. For identified proteins having multiple isoforms, the normalized volumes of all isoforms of a given protein were added together and statistical analysis was repeated on the totals.

To visualize the relationship of the different animals and treatment groups Principal Components Analysis (PCA) was performed by including all of the 454 matched spots. The first two principal components, which contained the largest variance (42.54% and 12.76%, respectively), allowed the best discrimination between the groups.

### Protein identification by mass spectrometry

For identification of spots, protein spots (2.0 mm diameter cores) were picked from picking gels using a robot-directed spot picker (Ettan spot picker, GE Healthcare). The spots selected for picking were determined on the basis of differential expression from the 2D-DIGE analysis. Some unchanged proteins were also picked for identification to create a map of the whole cell-free BAL proteome after depletion of the high abundance serum proteins. The picker head was calibrated using the reference stickers placed on the preparative picking gel and gel plugs were picked and placed in a bar-coded 96 well plate. All gel plugs were washed twice with 200 μl of 200 mM ammonium bicarbonate, 40% acetonitrile for 30 min at 37°C and dehydrated one time with 75% acetonitrile for 20 min followed by air drying. The protein was then digested with 20 μl of 0.02 μg/μl trypsin (Trypsin Gold, mass spectrometry grade, Promega Corporation, Madison, WI) overnight at 37°C. Fifty μl 0.1% trifluoroacetic acid (TFA), 50% acetonitrile was next added to each well and incubated for 30 min at 37°C. In-gel digested proteins were then transferred to 96-well extraction plates, dried by speed vac (Vaccufuge™ Concentrator, Eppendorf AG, Hamburg, Germany) and resuspended in 10 μl 0.5% TFA. Extracted protein/peptides were desalted and concentrated using C_18 _ZipTips (Millipore Corporation, Billerica, MA). Tips were wetted with 10 μl of 100% acetonitrile and equilibrated with 10 μl 0.1% TFA pH < 4. Samples were then drawn into ZipTip columns by aspirating for 7 cycles and then washed twice with 10 μl 0.1% TFA. Peptides were then eluted from the column with 5 μl of 0.1% TFA, 50% acetonitrile.

Peptides were analyzed by MALDI-ToF/ToF in the Mass Spectrometry Core at the Penn State University College of Medicine. A total of 2 μl of ZipTip cleaned samples (1 μl at a time) was applied onto a 384-well MALDI plate (Opti-TOF™ 384 Well Insert, Applied Biosystems, Foster City, CA) and then 0.7 μl of 2 mg/ml ACH cinnamic acid in 60:40 (acetonitrile:water) was spotted on each well containing peptide. All 13 calibration wells on the MALDI plate were spotted with (1:12 diluted) 4700 calibrant. Autolytic trypsin peptides were also used to internally calibrate the spectra to an accuracy of 20 ppm. Peptides were then analyzed by MALDI-ToF/ToF mass spectrometry using a 4800 Proteomics Analyzer (Applied Biosystems), calibrated with Applied Biosystems 4700 Proteomics Calibration Mix. For each sample, an initial mass spectrum was collected. Measurements were collected in the positive ion reflectron mode between 800 and 4000 m/z with a signal-to-noise filter of 10, mass exclusion tolerance of 0.2 Da, and a peak density filter of 50 peaks per 200 Da. Based on the initial mass spectrum, up to 15 precursors were selected for tandem mass spectrometry (MS/MS) analysis, excluding those included on an exclusion list containing trypsin autolysis, matrix, and tryptic peptides of human keratin, as well as those precursors identified in a "blank" gel plug. MS/MS was performed without collision-induced decay in a mass range from 60 Da to 20 Da below the precursor mass with a fragment tolerance of 0.2 Da for +1 charged ions. Using GPS Explorer 3.0 software (Applied Biosystems), the MS and MS/MS data were submitted to a MASCOT (v2.0.00) search engine for identification. The NCBI nonredundant database with the *Mus musculus *taxonomy (downloaded March 15, 2007, 107,758 entries searched) and a concatenated, reversed 'decoy' version were used for the searches with a mass accuracy of 50 ppm, 1 missed trypsin cleavage, fixed carbamidomethylation of cysteine residues and variable oxidation of methionine residues. A protein was considered identified if the MASCOT confidence interval was > 95th percentile (61 of 66 proteins were > 99 percentile) and those proteins with a MASCOT confidence interval < 95% were excluded from the subsequent analyses.

The PANTHER database and the scientific literature were used to assign molecular function and biological process to each identified protein, as well as to place each protein into the three major functional groups we defined (defense and immunity; redox regulation; protein modification and metabolism – see Results).

## Results

### Behavioral observations

Mice that were exposed to ozone behaved differently from those being exposed to filtered air. Soon after ozone exposure begins the fur becomes ruffled. After 30 minutes to 1 hour, the ozone-exposed mice become less active, curl up, and apparently sleep for the duration of the exposure period. Following the exposure, their activity returns to normal within the first hour. Mice exposed to filtered air are active throughout the exposure period. Both WT and KO mice behaved similarly during the ozone exposure period.

### BAL and cells

**Figure 1:**
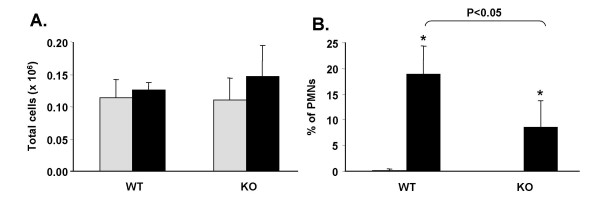
**Characteristics of BAL cells**. Following BAL the cells were removed by centrifugation and counted. Total cell numbers (A) were determined from an aliquot of cell suspension applied to a hemocytometer. Differential cell counts (B) were obtained by making cytocentrifuge preparations of BAL cells and doing differential cell counts. Gray bars represent FA-exposed samples and black bars represent ozone-exposed samples. Note that FA-exposed values are plotted in B, but values are very small (0.125% and 0% for WT and KO, respectively). Comparisons that were significantly different are indicated by a bracket and p-values.

The total number of cells recoverable in BAL fluid from WT and KO mice was similar (Figure 1), but there were statistically significant (p < 0.05) increases in the percentage of PMNs in ozone-exposed mice vs. FA-exposed mice. As would be expected, the increase in PMNs is mirrored by a statistically significant decrease in the number of monocytes/macrophages (not shown). The increase in PMNs in ozone-exposed KO mice was 50% less than that seen in WT mice (Figure 1). Total protein levels in the cell-free BAL were not measured, but in our previous study [8] using the same mouse strains and the same exposure conditions, there were no significant differences between strains of each group.

### Depletion of high abundance serum proteins from the mBAL

To enhance detection of most proteins in our proteomic studies we used a immunoaffinity system (MARS; Agilent – see Methods) to remove high abundance mouse serum proteins from the BAL. This, in turn, enables the loading of higher quantities of lower abundance BAL proteins. The MARS spin cartridge is designed to remove three high abundance proteins (albumin, IgG, and transferrin) from mouse serum and plasma. The removal of these proteins from BAL resulted in the removal of 85–90% of total mass of BAL protein, with about 10–15% of the total protein applied to the column emerging in the flow-through fractions. Similar recovery in terms of protein content in the flow-through fraction was observed in all samples under study. The percentage recoveries of the proteins were determined by micro BCA protein assay. The removal of 80–90% of the albumin and transferrin was confirmed by 2-D gel analysis (not shown). This selective immunodepletion allows an enriched pool of the lower abundance protein to be loaded on gels.

### Overview of 2D-DIGE results

**Figure 2:**
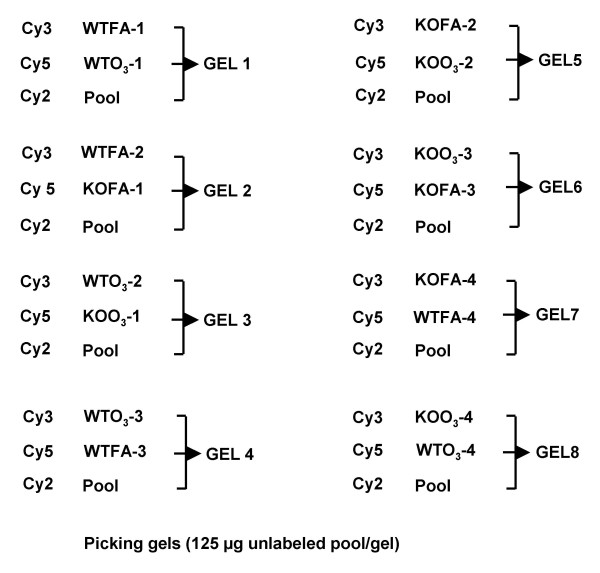
**Sample layout for 2D-DIGE**. The arrangement of individual samples for staining with either Cy3 or Cy5 and their layout on the gels is presented diagrammatically. Note that for each group of 4 samples two were stained with Cy3 and two with Cy5. An aliquot of a Cy2-labeled pool is included on all gels.

Immunodepleted BAL samples from WT and KO mice following exposure to filtered air or ozone (n = 4 for each of the groups under study) were subjected to 2D-DIGE and analyzed with Progenesis SameSpots. The arrangement of samples on the gels is shown in Figure 2, including the switching of samples between Cy3 and Cy5 to prevent any potential dye bias. We were able to match a total of 454 protein spots in all of the samples.

#### Principal components analysis

**Figure 3:**
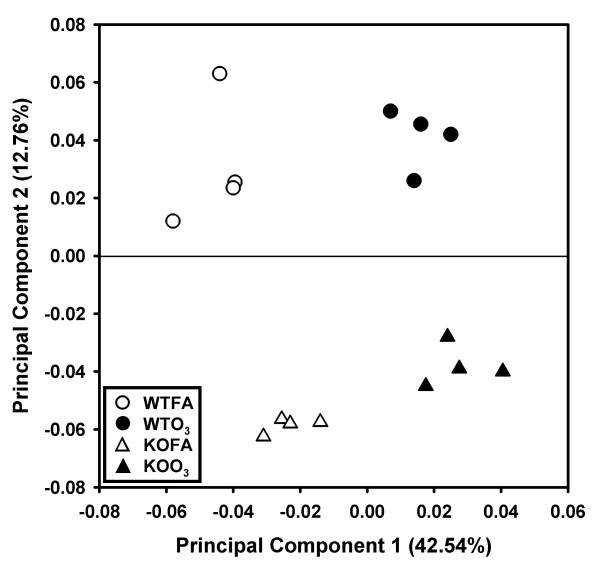
**Principal component analysis (PCA) of 2D-DIGE data**. This figure depicts a principal component analysis in which principal components (PC) 1 and 2 are plotted for all 454 matched protein spots. PC1 accounted for 42.54% of the variance and PC2 accounted for 12.76% and segregated the groups by ozone exposure and SP-A deletion, respectively. Circles represent the WT mice and triangles represent KO mice. The open symbols represent samples that were exposed to FA and the black symbols represent those exposed to ozone.

To examine the relationship of the samples in each group as well as the groups to each other based on the proteomic profile, principal components analysis of the data (Figure 3) was performed using all 454 matched protein spots. Principal components 1 and 2 accounted for 42.54% and 12.76% of the study variance, respectively. Principal component 1 segregated the samples by ozone exposure and principal component 2 by strain. The contributions to the relative variance of the two principal components (42.54% vs 12.76%) indicate that the effect of ozone exposure (principal component 1) on changes in the BAL proteome is greater than that of SP-A deletion (principal component 2). Each of the 16 independent animals (4/group) represented by the markers in the figure represents the combined weighted average of the first two principal components for each of the 454 protein spots. Each of the four experimental groups are tightly clustered with no overlap among groups.

#### Identification of proteins

**Figure 4:**
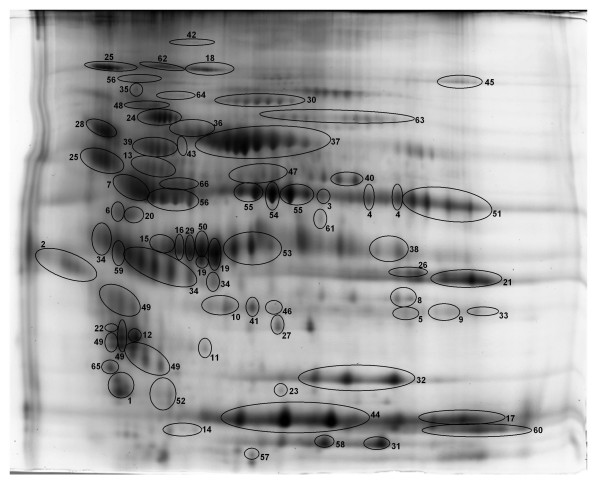
**Identified proteins on the reference gel**. An image of the reference gel is shown in which all identified proteins are circled and numbered. The names of each protein and their functions are given in Additional File 3. Note that for this study the normalized volumes for all isoforms of a given protein were added together and the sum was used for statistical comparisons.

Next, we picked many of the 454 spots detected and subjected them to analysis with tandem mass spectrometry. In this report we have restricted our list of proteins identified by MALDI-ToF/ToF to those spots with MASCOT confidence interval scores of > 95%. This resulted in the identification of 66 proteins made up of 141 protein spots, of which the spots identifying 61 proteins had protein identifications with > 99% MASCOT confidence intervals. More than half of the identified proteins were represented in multiple isoforms/spots. If the normalized volumes of the identified spots are expressed as a percentage of the total normalized volume for all spots, the identified proteins account for 55% of the expressed protein detected on the gel. The identified proteins are circled, numbered, and shown in Figure 4. Additional File 3 lists all of the identified proteins, their accession numbers, and the molecular functions and biological processes assigned to each in the PANTHER database. The antecedents for each of the abbreviated molecular functions and biological processes as well as reference(s) for these functions or processes are included in the legend for Additional File 3. All subsequent analyses were restricted to the 66 proteins that were identified by MALDI-ToF/ToF. For these analyses the values for the normalized volumes for all of the protein spots (isoforms) making up each identified protein were added together to obtain a total for each protein. Statistical analyses were then done using these values.

#### Assignment of identified proteins into groups

By using gene ontology resources, such as the PANTHER database, and conventional searches of the literature we were able to assign many of the identified proteins to three major "functional groups" which are indicated in Additional File 3, along with supporting references [30,52,55-100].

The largest functional group of proteins (n = 30) we identified were those involved in defense and immunity functions (DEF) (Table 1). This is a diverse group of proteins that includes proteins that play a role in defense against pathogens, others that have been implicated in the regulation of inflammatory processes, and others that participate in the detoxification of toxins or other potentially noxious substances. The diversity of this group is necessitated by the extremely diverse array of potential insults, including pathogens and toxins, which the lung is exposed to during ventilation. To contend with this diversity, the lung employs an assortment of mechanisms.

**Table 1:** Defense and immunity proteins

		Normalized Volumes Mean (SD)		WTFA vs WTO_3_ p ≤ 0.05	Normalized Volumes Mean (SD)		KOFA vs KOO_3_ p ≤ 0.05	WTFA vs KOFA p ≤ 0.05	WTO_3 _vs KOO_3_ p ≤ 0.05
		WTFA	WTO_3_		KOFA	KOO_3_			
Adipsin (complement factor D)	↑	2.05 (0.337)	2.43 (0.610)		2.13 (0.742)	2.34 (0.655)			
Alpha-1-antitrypsin 1-1	↓	1.18 (0.191)	1.04 (0.102)		1.35 (0.369)	0.94 (0.210)			
Alpha 1-antitrypsin 1-6	↑	2.15 (.230)	2.94 (0.353)	*	2.85 (0.484)	3.47 (0.712)		*	
Annexin A1	↓	3.05 (0.168)	2.57 (0.381)	**.058**	2.73 (0.409)	2.38 (0.517)			
Annexin A5	↑	1.10 (0.155)	1.30 (0.251)		0.58 (0.078)	1.04 (0.171)	*	*	
Antithrombin-III	↑	3.08 (0.589)	4.54 (0.659)	*	3.53 (0.342)	4.78 (0.454)	*		
Apolipoprotein A-I	↑	1.59 (0.300)	2.18 (0.350)	*	2.24 (0.668)	4.41 (1.038)	*		*
Chain A, Mammalian Lectin Ym1	↓	3.78 (1.000)	2.76 (0.290)		3.03 (0.053)	1.97 (0.903)	**.058**		
Chia protein	↓	1.20 (0.244)	0.88 (0.172)	**.075**	1.49 (0.367)	0.82 (0.354)	*		
Chitinase-3-like protein 1 precursor (GP-39)	↓	11.19 (1.860)	7.35 (1.959)	*	9.26 (2.089)	4.46 (1.843)	*		
Complement component C3	↑	0.65 (0.142)	1.04 (0.212)	*	0.74 (0.248)	1.24 (0.261)	*		
Complement component C5	↓	7.26 (0.617)	6.88 (0.999)		7.32 (0.692)	6.08 (0.766)	**.053**		
Esterase 1	↑	2.73 (0.187)	3.85 (0.960)	*	3.31 (0.220)	4.44 (0.687)	*	*	
Ferritin (light chain)	↓	1.43 (0.325)	1.21 (0.180)		1.34 (0.382)	1.13 (0.380)			
Gluthathione S-transferase A4 (GST A4-4)	↓	1.41 (0.153)	0.80 (0.445)	*	1.32 (0.148)	0.65 (0.146)	*		
Glutathione S-transferase omega 1	↓	3.52 (0.140)	2.78 (0.416)	*	2.94 (0.422)	2.77 (0.986)		*	*
Haptoglobin	↓	12.71 (2.040)	11.21 (0.835)		11.47 (0.326)	7.61 (0.764)	*		
Heat shock protein 1, alpha	↑	0.75 (0.092)	0.95 (0.114)	*	0.70 (0.164)	0.95 (0.076)	*		
Heat shock protein 70	↑	3.93 (0.807)	5.82 (1.173)	*	4.64 (0.556)	7.79 (1.026)	*		
Kininogen 1	↑	5.36 (1.723)	6.99 (0.703)		5.89 (0.514)	7.65 (0.456)	*		
Lactate dehydrogenase 2, B chain	↓	1.26 (0.417)	1.03 (0.520)		1.05 (0.179)	0.84 (0.062)	**.072**		
Murinoglobulin-1 precursor (MuG1)	↑	0.77 (0.166)	1.07 (0.136)	*	0.88 (0.073)	1.26 (0.131)	*		
Peroxiredoxin 6 (Antioxidant protein 2)	↓	5.60 (0.884)	3.35 (1.694)	**.057**	5.39 (0.486)	3.11 (0.667)	*		
Plasminogen	↓	8.39 (1.194)	7.35 (0.653)		8.82 (0.775)	7.70 (2.054)			
Pregnancy zone protein	↑	0.76 (0.166)	0.83 (0.059)		0.94 (0.343)	1.05 (0.080)			
Prothrombin precursor (EC 3.4.21.5)	↑	1.42 (0.171)	2.01 (0.423)	*	1.56 (0.167)	2.15 (0.301)	*		
Pulmonary surfactant-assoc. protein A (SP-A)	↓	36.21 (7.512)	29.07 (6.055)		-	'-		*	
Selenium binding protein 1	↓	1.42 (0.177)	0.83 (0.402)	*	1.39 (0.212)	0.68 (0.105)	*		
Selenium binding protein 2	↓	5.61 (0.794)	3.53 (1.407)	*	5.33 (0.742)	3.00 (0.472)	*		
Similar to Glutathione S-transferase Ya chain	↓	1.53 (0.166)	0.94 (0.443)	*	1.43 (0.174)	0.83 (0.216)	*		

The proteins assigned to the defense and immunity category (DEF) are listed (n = 30). An arrow indicates whether levels of expression are increased (↑) or decreased (↓) in response to ozone. The mean values for the normalized volumes and standard deviations for each named protein are given for WTFA, WTO_3_, KOFA, and KOO_3_. The asterisk indicates whether the p-value for the comparisons of WTFA vs. WTO3; KOFA vs. KOO_3_; WTFA vs. KOFA; and WTO_3 _vs. KOO_3 _was ≤ 0.05. Values that were slightly above 0.05 are also given.

A second major functional group consisted of proteins (n = 22) playing a role in the regulation of redox balance in the lung (RED) (Table 2). These include proteins generating reactive oxygen and nitrogen species (RONS), neutralizing RONS, and proteins binding molecules such as iron, copper, and heme that are involved in processes related to redox balance. This diversity of proteins is essential in the oxidative environment of the lung where there are high oxygen levels and where host defense elements are constantly dealing with inhaled pathogenic and toxic threats with mechanisms that can generate RONS.

**Table 2:** Proteins involved in redox balance

		Normalized Volumes Mean (SD)		WTFA vs WTO_3_ p ≤ 0.05	Normalized Volumes Mean (SD)		KOFA vs KOO_3_ p ≤ 0.05	WTFA vs KOFA p ≤ 0.05	WTO_3 _vs KOO_3_ p ≤ 0.05
		WTFA	WTO_3_		KOFA	KOO_3_			
Aldehyde dehydrogenase AHD-M1	↓	1.24 (0.163)	0.93 (0.286)		1.23 (0.163)	0.86 (0.140)	*		
Aldehyde dehydrogenase (EC 1.2.1.5)	↓	2.75 (0.471)	1.83 (0.570)	*	2.57 (0.365)	1.68 (0.362)	*		
Aldose reductase (EC 1.1.1.21)	↓	1.84 (0.193)	1.60 (0.294)		1.88 (0.362)	1.53 (0.348)			
Apolipoprotein A-I	↑	1.59 (0.300)	2.18 (0.350)	*	2.24 (0.668)	4.41 (1.038)	*		*
Apolipooprolein A-IV	↑	2.50 (0.221)	3.01 (0.306)	*	2.69 (0.122)	3.27 (0.611)			
Carbonyl reductase 2	↓	4.47 (0.537)	2.66 (1.329)	*	4.67 (0.543)	2.36 (0.453)	*		
Ceruloplasmin isoform	↑	7.51 (0.766)	9.48 (1.487)	**.056**	7.53 (0.960)	9.39 (1.337)	**.065**		
Cytosolic malate dehydrogenase	↓	1.18 (0.126)	0.80 (0.234)	*	1.09 (0.141)	0.80 (0.124)	*		
Ferritin light chain	↓	1.43 (0.325)	1.21 (0.180)		1.34 (0.382)	1.13 (0.380)			
Gelsolin	↑	7.07 (1.072)	11.69 (3.636)	**.051**	6.87 (0.347)	11.89 (1.682)	*		
Glutathione S-transferase A4 (GST A4-4)	↓	1.41 (0.153)	0.80 (0.445)	*	1.32 (0.148)	0.65 (0.146)	*		
Glutathione S-transferase omega 1	↓	3.52 (0.140)	2.78 (0.416)	*	2.94 (0.422)	2.77 (0.986)		*	
Glyceraldehyde-3-phosphate dehydrogenase (EC 1.2.1.12)	↓	2.54 (0.750)	1.94 (1.125)		1.99 (0.379)	1.48 (0.241)	**.067**		
Heat shock protein 70	↑	3.93 (0.807)	5.82 (1.173)	*	4.64 (0.556)	7.79 (1.026)	*		*
Hemopexin	↑	9.19 (1.184)	10.85 (1.000)	**.076**	11.49 (0.759)	13.71 (0.905)	*	*	*
Isocitrate dehydrogenase cytoplasmic (EC 1.1.1.42)	↑	2.14 (0.119)	2.12 (0.152)		1.64 (0.241)	1.97 (0.662)		*	
Lactate dehydrogenase 2, B chain	↓	1.26 (0.417)	1.03 (0.520)	*	1.05 (0.179)	0.84 (0.062)	**.072**		
Peroxiredoxin 6 (Antioxidant protein 2; AOP2)	↓	5.60 (0.884)	3.35 (1.694)	**.057**	5.39 (0.486)	3.11 (0.667)	*		
Protein disulfide-isomerase A3 (EC 5.3.4.1)	↓	5.62 (1.658)	4.87 (1.685)		4.67 (0.956)	4.64 (0.211)			
Retinal dehydrogenase (RALDH1)(EC 1.2.1.36)	↓	7.88 (1.629)	5.55 (1.817)		7.85 (1.118)	5.07 (1.220)	*		
Selenium binding protein 1	↓	1.42 (0.177)	0.83 (0.402)	*	1.39 (0.212)	0.68 (0.105)	*		
Selenium binding protein 2	↓	5.61 (0.794)	3.53 (1.407)	*	5.33 (0.742)	3.00 (0.472)	*		

The proteins assigned to the redox balance category (RED) are listed (n = 22). An arrow indicates whether levels of expression are increased (↑) or decreased (↓) in response to ozone. The mean values for the normalized volumes and standard deviations for each named protein are given for WTFA, WTO_3_, KOFA, and KOO_3_. The asterisk indicates whether the p-value for the comparisons of WTFA vs. WTO_3_; KOFA vs. KOO_3_; WTFA vs. KOFA; and WTO_3 _vs. KOO_3 _was ≤ 0.05. Values that were slightly above 0.05 are also given.

The third major functional group defined consisted of proteins (n = 18) that we broadly categorized as being involved in protein metabolism and modification, including proteins with chaperone function (PMM) (Table 3). This group included a number of proteases and antiproteases, as well as proteins such as several chaperones, which are involved in the metabolism of proteins that have been modified in various ways, including oxidative modifications.

**Table 3:** Proteins involved in protein modification and metabolism

		Normalized Volumes Mean (SD)		WTFA vs WTO_3_ p ≤ 0.05	Normalized Volumes Mean (SD)		KOFA vs KOO_3_ p ≤ 0.05	WTFA vs KOFA p ≤ 0.05	WTO_3 _vs KOO_3_ p ≤ 0.05
		WTFA	WTO_3_		KOFA	KOO_3_			
14-3-3 zeta	↓ ↑	3.75 (0.425)	3.56 (0.285)		4.21 (0.329)	4.39 (0.552)			*
Adipsin (complement factor D)	↑	2.05 (0.337)	2.43 (0.610)		2.13 (0.472)	2.34 (0.655)			
Alpha-1-antitrypsin 1-1 precursor	↓	1.18 (0.191)	1.04 (0.102)		1.35 (0.369)	0.94 (0.210)			
Alpha-1-antitrypsin 1-6 precursor	↑	2.15 (0.230)	2.94 (0.353)	*	2.85 (0.484)	3.47 (0.712)		*	
Antithrombin-III	↑	3.08 (0.589)	4.54 (0.659)	*	3.53 (0.342)	4.78 (0.454)	*		
Contrapsin	↑	8.72 (0.626)	13.00 (3.512)	**.053**	10.28 (1.886)	13.47 (2.994)			
Haptoglobin	↓	12.71 (2.040)	11.21 (0.835)		11.47 (0.326)	7.61 (0.764)	*		*
Heat shock protein 1, alpha	↑	0.75 (0.092)	0.95 (0.114)	*	0.70 (0.164)	0.95 (0.076)	*		
Heat shock protein 70	↑	3.93 (0.807)	5.82 (1.173)	*	4.64 (0.556)	7.79 (1.026)	*		*
Kininogen1	↑	5.36 (1.723)	6.99 (0.703)		5.89 (0.514)	7.65 (0.456)	*		
Murinoglobulin-1 precursor (MuG1)	↑	0.77 (0.166)	1.07 (0.136)	*	0.88 (0.073)	1.26 (0.131)	*		**.08**
Plasminogen	↓	8.39 (1.194)	7.35 (0.653)		8.82 (0.775)	7.70 (2.054)			
Pregnancy zone protein	↑	0.76 (0.166)	0.83 (0.059)		0.94 (0.343)	1.05 (0.080)			*
Protein disulfide-isomerase A3 precursor	↓	5.62 (1.658)	4.87 (1.685)		4.67 (0.956)	4.64 (0.211)			
Prothrombin precursor (EC 3.4.21.5)	↑	1.42 (0.171)	2.01 (0.423)	*	1.56 (0.167)	2.15 (0.301)	*		
Serine (or cysteine) proteinase inhibitor, clade A, member 1e	↑	5.34 (0.842)	7.41 (0.723)	*	6.10 (0.636)	8.34 (0.800)	*		
Transitional endoplasmic reticulum ATPase	↑	1.62 (0.230)	2.94 (1.149)	**.066**	1.92 (0.469)	3.91 (0.527)	*		
Tyr 3-monooxygenase/trp 5-monooxygenase activation protein	↑	0.87 (0.059)	1.04 (0.092)	*	0.93 (0.133)	1.24 (0.135)	*		**.054**

The proteins assigned to the protein modification and metabolism and chaperone category (PMM) are listed (n = 18). An arrow indicates whether levels of expression are increased (↑) or decreased (↓) in response to ozone. The mean values for the normalized volumes for each named protein are given for WTFA, WTO_3_, KOFA, and KOO_3_. The asterisk indicates whether the p-value for the comparisons of WTFA vs. WTO_3_; KOFA vs. KOO_3_; WTFA vs. KOFA; and WTO_3 _vs. KOO_3 _was ≤ 0.05. Values that were slightly above 0.05 are also given.

A number of proteins are included in more than one of the three groups, such as heat shock protein 70, which in addition to its role as a chaperone, can help regulate cellular redox status, and may serve an anti-inflammatory role by limiting the proliferation of certain cell types [52,87,88]; the glutathione S-transferases, which are classified as immunity and defense proteins by PANTHER, in addition to their functions in redox regulation [79,80]; and pregnancy zone protein, which is a proteinase inhibitor, but plays a role in defense and immunity by modulating T-cell activation [96].

### Changes in the expression of specific protein groups

Thirty proteins were assigned to the defense and immunity (DEF) group (29 excluding SP-A). Significant differences in the levels of expression between filtered air-exposed WT and KO mice (Table 1; WTFA vs KOFA) were observed in 4 of the 30 proteins (excluding SP-A which is absent in KO mice) and between ozone-exposed WT and KO mice in 2 proteins (Table 1; WTO_3 _vs. KOO_3_). In response to ozone there were increases in 13 proteins and decreases in 17. Significant changes in 15 of these proteins (50%) occurred in WT mice (Table 1; WTFA vs. WTO_3_). A comparison of KOFA and KOO_3 _mice showed an increased number of responses with significant changes in response to ozone in 18 (60%) of these proteins. Of the 29 proteins (excluding SP-A) expressed in both mouse strains, the % change [*(ozone-exposed - FA-exposed) ÷ FA-exposed = % change*] in response to ozone was greater in the KO mice in 21 (72%) of them.

The redox balance group (RED) of proteins (Table 2) contained 22 entries, including 15 ozone-induced decreases and 7 increases. Three proteins differed significantly in their levels between filtered air-exposed WT and KO mice (Table 2; WTFA vs KOFA) and 3 proteins differed significantly between ozone-exposed WT and KO mice (Table 2; WTO_3 _vs KOO_3_). In WTFA mice compared to WTO_3 _there were 11 proteins that underwent significant changes, and in KOFA mice there were 12 proteins compared to KOO_3 _mice undergoing significant changes. Of the 22 proteins in the RED group, the % change in response to ozone was greater in 18 proteins (82%) in the KO mice compared to WT mice.

We categorized 18 proteins as being involved in protein modification and metabolism or chaperone (PMM) function (Table 3). Most of the ozone-induced changes (14 proteins) observed were increases in levels of expression. There was 1 protein in which there was a significant difference between the FA-exposed mice in both strains (Table 3; WTFA vs. KOFA) and 4 proteins that differed significantly when ozone-exposed WT and KO mice were compared (Table 3; WTO_3 _vs KOO_3_). The ozone-induced changes were statistically significant for 8 proteins in the WT mice (WTFA vs WTO_3_) and 10 in the KO mice (KOFA vs KOO_3_). In the PMM group of proteins there were 10 proteins (56%) where the % change in response to ozone exposure was greater in the KO mice than in WT mice.

### Overview of strain differences in protein expression and response to ozone

After examining changes in the individual functional groups we re-evaluated these data by looking at the overall response pattern of the 64 proteins (excluding SP-A and transferrin). It was notable that, excluding SP-A, only 9 proteins varied significantly between WT and KO mice exposed to filtered air and that most of these differences were rather modest. These changes in the three functional groups are indicated in the WTFA vs. KOFA columns in Tables 1, 2, and 3. A total of 11 significant changes were found when we compared ozone-exposed WT to KO mice (see Tables 1, 2, and 3; WTO_3 _vs KOO_3_).

Looking at changes resulting from ozone exposure in the WT mice 25 (of 64) proteins (39%) differed significantly, whereas in the KO mice 37 proteins (58%) were significantly changed. Not only were there more significant changes in the KO mice, but the percent change in the KO mice was greater for 42 (66%) of the observed changes (significant and not significant) than for the WT mice. It is also noteworthy that when comparing the 64 proteins (excluding SP-A and transferrin), in the majority of cases (70% vs. 30%) the % change in the response to ozone (Table 4; KO > WT) was of greater magnitude in the KO mice than in the WT mice. It is also interesting to note that in all three of the functional protein groups (DEF, RED, and PMM) described above, the changes in ozone-exposed mice compared to FA-exposed mice were greater in the KO mice than in the WT mice. This trend was particularly pronounced: a) in the DEF group of proteins where 21 (72%) of the 29 changing proteins underwent greater changes in the KO mice than in the WT; and b) in the RED group where 18 (82%) of the 22 changes were greater in the KO mice (Table 4; KO > WT).

**Table 4:** Summary of responses

	Total	O3↑	O3↓	WT*	KO*	KO > WT	WT > KO
Overall	64	-	-	25	37	45 (70%)	19 (30%)
DEF (excluding SP-A)	29	13	16	15	18	21 (72%)	8 (28%)
RED	22	7	15	11	13	18 (82%)	4 (18%)
PMM	18^#^	14	5	8	10	10 (56%)	8 (44%)

An overview of the changes in expression in response to ozone is given. The number of proteins in the entire data set and in each of the three functional groups is given. The number of responses to ozone that involved an increase in levels of expression (O_3_↑) or a decrease (O_3_↓) is indicated. Note (#) that after ozone exposure one protein in the PMM group is decreased in WT mice and increased in KO. The number of significant changes in comparisons of WTFA vs. WTO_3 _(WT*) or KOFA vs. KOO_3 _(KO*) is indicated. For each group, the number of ozone responses where the magnitude is greater in KO mice is given (KO > WT), as are those where WT is greater (WT > KO).

In many of the proteins showing a change from one group to another a common pattern was observed. This pattern is characterized by: a) levels of expression in KOFA mice being closer to WTO_3 _mice than to WTFA mice; and b) by responses to ozone in the KOO_3 _mice that result in increases or decreases (depending on the particular protein) in expression levels of a certain protein exceeding those in the WTO_3 _mice.

### Changes in specific proteins

**Figure 5:**
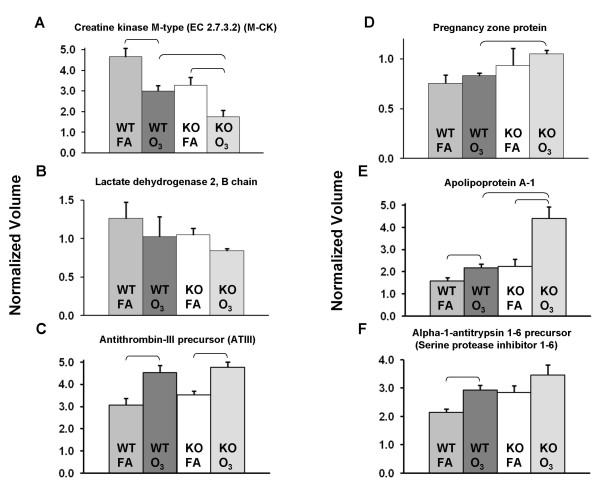
**Representative graphs of specific protein changes**. Histograms depicting levels of specific expressed proteins from WT (WTFA, WTO_3_) and KO (KOFA, KOO_3_) mice after exposure to FA or ozone (O_3_) are shown +/- standard deviation. The graphs depict data from: A) creatine kinase M-type; B) lactate dehyrogenase 2; C) antithrombin III; D) pregnancy zone protein; E) apolipoprotein A-I; and F) alpha-1-antitrypsin 1–6. Comparisons between groups that were statistically significant (p < 0.05) are indicated with a bracket.

Several examples that illustrate the trends described above are shown in Figure 5. The corresponding normalized volumes for most of these proteins are given in Tables 1, 2, and 3. In Figure 5 Panels A and B a reduction in levels of expression of creatine kinase M-type and lactate dehydrogenase 2, respectively, was observed in WTO_3 _mice. The KOFA mice have levels that are similar to the WTO_3 _mice but reductions are observed in KOO_3_compared to KOFA or WTO_3_. Panels C through F show examples (including respectively, antithrombin III, pregnancy zone protein, apolipoprotein A-1, and alpha-1-antitrypsin 1–6) of ozone-induced increases in WTO_3 _mice that are mirrored by similar or greater increased levels of expression in KOFA mice. The levels of these proteins in KOO_3 _mice are further increased to a varying degree compared to WTO_3 _or KOFA. Apolipoprotein A-1 has a role in defense and immunity by its ability to bind and neutralize LPS and in redox regulation by its role in neutralizing lipid hydroperoxides and decreasing neutrophil degranulation and superoxide production. Pregnancy zone protein is an antiprotease, but has also been shown to have anti-inflammatory activity [96]. Antithrombin III and alpha-1-antitrypsin 1–6 have antiprotease activity and have been shown to have anti-inflammatory activities [62,64].

In these examples and a number of others (not shown), a consistent trend for the levels of a particular protein is observed. A progressive increase (or in some cases decrease) is observed as one progresses from WTFA to WTO_3 _to KOFA to KOO_3_. Indeed, in roughly two-thirds of the proteins listed in Tables 1, 2, and 3 KOFA values differed from WTFA values in the same manner (increasing or decreasing) as WTO_3 _differed from WTFA. However, there were only 6 cases (not including SP-A) where these differences between WTFA and KOFA achieved statistical significance. A similar situation was observed when we compared WTO_3 _to KOO_3 _values where values for a given protein followed this progression, but differences were only significant in a few cases. The similarity of expression patterns between WTO_3 _mice and KOFA mice supports the possibility that an increase in oxidative stress in KOFA mice exists, perhaps due to the absence of SP-A, an innate immune protein known to have antioxidant activity.

## Discussion

Ozone and other air pollutants are known to cause lung inflammation, to exacerbate other lung diseases such as asthma, and to increase susceptibility to infections. The mechanism(s) behind these effects are not well understood but may involve proteins in the epithelial lining fluid of the lung that have a role in innate immune mechanisms. One of these proteins, SP-A, is involved in many aspects of innate immunity. A number of studies have described disruptions in SP-A function following exposure to ozone or other oxidants and others have presented evidence indicating that SP-A may have antioxidant function. In several previous studies we have compared the responses of WT and KO mice to ozone exposure and their relative susceptibility to infection after ozone exposure [8,9,16]. We found that KO mice sustained greater tissue damage after ozone exposure and were more susceptible to infection. These results indicated that SP-A may play a role in protecting the lung from oxidant-induced injury and from infection. However, the basis for these differences was unclear.

In this study we sought to build upon and extend the existing information. In order to gain insight into the responsible mechanisms we employed a discovery proteomics approach to characterize changes in the expression of proteins in mouse BAL following ozone exposure and assess the contribution of SP-A to this response by comparing the BAL proteomes of SP-A KO mice and WT mice for the first time and comparing the responses of these two mouse strains to ozone exposure. Using the PANTHER ontology database and the published literature, the proteins identified via MALDI-ToF/ToF MS were assigned to three major functional groups. This broad categorization may provide a more informative overview than the dozens of different biological processes and molecular functions assigned by PANTHER alone. Subsequent analysis compared significant changes among the experimental groups (WTFA, WTO_3_, KOFA, KOO_3_) and enabled us to postulate an important role for SP-A in response to ozone-induced oxidative stress. This putative role (i.e. its involvement in redox regulation, in addition to its well-described role in innate immunity) builds on several reports that have described an antioxidant function for SP-A [101-103].

When we compared the responses of WT and SP-A KO mice to oxidative stress (in the form of an acute ozone exposure), we identified a number of changes in protein expression. These were consistent with oxidative stress and were associated with known complications of ozone exposure, including increased susceptibility to infection in humans [1,18,19] and animals [1,9,16]. In addition, we observed that the responses to ozone, in terms of percent change, were often more pronounced in KOO_3 _compared to WTO_3 _mice, indicating that KO mice may be more susceptible to ozone-induced oxidative stress. This observation is consistent with our earlier study in which we reported increased BAL levels of LDH in KO mice, indicating that KO mice sustained more ozone-induced tissue damage than WT mice [8]. Moreover, in a number of cases we found that control KOFA mice expressed many proteins at levels equivalent to, or even exceeding, WTO_3_, indicating that KO mice may be subject to oxidative stress, even in the absence of the exogenous ozone-induced oxidative stress. We speculate that this increase occurs because of the lack of SP-A, an important host defense protein that plays an antioxidant or oxidant scavenger role in the alveolus. This is based on several converging lines of evidence including: 1) the attribution of an antioxidant role to SP-A [101-103]; 2) the demonstration that SP-A is highly susceptible to oxidative modification by carbonylation)[53] and to ozone-induced oxidation of methionine residues [34], and that its function is disrupted by these oxidative modifications)[9,31-34]; and 3) the description of other systems in which proteins serve as "sacrificial antioxidants" [104-109].

In past studies we and other investigators have targeted specific proteins in the characterization of the ozone response. The selection of target proteins was based on previously published studies and tended to provide incremental advances in our understanding of the involved mechanisms. A case in point is the analysis of cytokines and chemokines that may be involved in ozone-induced inflammation [8,40-44]. Studies of this type have only examined a handful of the dozens of cytokines that may potentially play a role in this process. Furthermore, the functional redundancy of some of these molecules can complicate interpretation.

The two-dimensional electrophoretic analysis of rodent BAL proteins after ozone exposure has been very limited. One preliminary study has used conventional 2-D gel approaches to examine differences in BAL protein expression between an ozone-sensitive strain of mice and an ozone-resistant strain [46], although these authors did not examine ozone-induced changes. Interestingly, one of the proteins they found to differ between strains, was peroxiredoxin 6 (identified in their study as antioxidant protein 2) which we found to be significantly reduced after ozone exposure in both strains that we studied. The other protein that differed between strains in their study, Clara cell protein 10, was too small to be resolved in the second dimensional gel system we used. Another study with rats examined the effect of prior ozone exposure on 1-nitronaphthalene adduction of BAL proteins [45] and found peroxiredoxin 6 to be increasingly adducted following ozone exposure. By applying a two-dimensional gel-based discovery proteomics approach to the study of ozone exposure we hoped to obtain additional information about the role of molecules such as peroxiredoxin 6 in this process and to identify previously overlooked molecules that may also play important roles, thereby gaining insight into the interplay of different processes affected by ozone exposure and the resulting pathophysiology. Moreover, the ability (via the use of a normalization pool) in 2D-DIGE to internally standardize the protein spots of all BAL samples in all gels under study provides a major advance that previous BAL studies largely lacked.

### Proteome of WT mice

In our previous study examining the effects of ozone exposure on mice [8] we reported that SP-A, a protein that is highly susceptible to oxidation, was oxidized immediately after ozone exposure, whereas increases in total protein oxidation were not detectable until 4 hours later. This delayed oxidation coincides with an influx of neutrophils into the alveolar space that may be a consequence of their activation by ozone-induced tissue damage and the subsequent production of RONS by these cells. In the present study, most of the significant changes in levels of expression of the RED protein group involved in redox balance were decreases, a finding that would be consistent with increased degradation of proteins that had been oxidatively modified while neutralizing reactive oxidants [110]. On the other hand, in the PMM group of proteins with roles in protein metabolism and modification and the chaperones, half of these proteins changed significantly with most undergoing increases after ozone exposure. One could speculate that this is a response to the increased oxidative modification of proteins and the apparent increased turnover of the proteins involved in regulating redox balance. Thus, the data from this discovery proteomics study, together with previously published data [8], support the postulate that in response to ozone-induced oxidative stress there is an increase in total protein oxidation and this reflects decreases in proteins involved in redox balance and increases in proteins involved in protein modification and metabolism.

Approximately half of the DEF group of defense and immunity proteins underwent significant changes, with changes that included roughly equal numbers of increases and decreases. This "mixed" response may explain why ozone exposure has been reported to prime pulmonary innate immunity, and thereby enhance the response to LPS [111], while impairing clearance of pathogens that are dependent upon removal by cell-mediated immune mechanisms, including *Listeria monocytogenes *or *Klebsiella pneumoniae *[9,112]. There is precedent for selective changes in susceptibility. Published studies have shown that genetic ablation of SP-A increases the susceptibility of the SP-A KO mouse to organisms whose recognition and clearance are highly dependent on SP-A, such as group B streptococcus and *Pseudomonas aeruginosa *[35,36]. On the other hand, increased levels of SP-A can predispose the host to organisms, such as *Pneumocystis carinii *[113,114], that are typically cleared by other mechanisms. Taken together, these responses document ozone-induced changes in several dozen BAL proteins, many of which had not been previously examined in this context.

### Comparison of WT and KO mice

Although an analysis of the ozone response revealed an overall similar response between WTO_3 _and KOO_3_, some differences were also observed. One striking difference between the WT and SP-A KO mice was in the DEF and RED protein groups, where roughly three-fourths of the responses were greater in the KO mice. In most cases the significant ozone-induced changes in the KOO_3 _mice were similar to trends observed in the WTO_3 _mice, but the magnitude of the change was greater in the KOO_3 _mice than in WTO_3_. This is also exemplified by the PCA analysis in which the first principal component, which separated groups by ozone exposure, accounted for a greater degree of study variance (42.54%) than the second component (12.76% of study variance) which segregated KO mice from WT. This indicates both an increased sensitivity to the oxidative stress caused by ozone exposure in both WT and KO, and a more vigorous and perhaps less well-regulated response to the ozone exposure in KO mice.

Comparison of the values of KOFA mice with the WTFA and WTO_3 _values revealed another very interesting point. In many cases the baseline (FA-exposed) value of the KOFA mice differed from the WTFA values in a similar fashion as the WTO_3 _mice differed from the WTFA group. For example, lactate dehydrogenase and sec14-like 3 levels were reduced after ozone exposure and the corresponding levels in the KOFA group were similar to the WTO_3 _group. Following ozone exposure, the levels in the KOO_3 _mice were further reduced. Similarly, there were a number of cases where increases in WTO_3 _mice were mirrored by similar increases in the KOFA mice that were subsequently further increased by ozone as assessed by the values observed in KOO_3 _mice. These include apolipoprotein A-I, kininogen 1, and pregnancy zone protein, among others. The similarity between the levels of many proteins in the KOFA mice to those seen in WTO_3 _mice (in other words, baseline levels in the KO mice corresponded to levels induced by oxidative stress in the WT mice) led us to propose the following scenario.

Many of the changes in WTO_3 _mice are likely due to oxidative stress resulting from acute ozone exposure. We have demonstrated that SP-A is highly susceptible to oxidative modification and that its modification significantly compromises its function))[31-33,53,115-117]. In WT mice SP-A is an abundant BAL protein and several lines of evidence have linked it to redox regulation and led investigators to propose an antioxidant function for SP-A. In these papers it has been demonstrated that SP-A inhibits lipid peroxidation [101,102,116] and that it can restore function to oxidized surfactant [103]. We postulate that the reason that many proteins in the KOFA mice have levels similar to WTO_3 _mice is because the KOFA mice are under chronic oxidative stress due to the lack of SP-A. In our previous study of ozone exposure and SP-A KO mice we did not detect differences in glutathione levels between WT and KO mice, but we did not measure levels of the many other enzymatic and non-enzymatic antioxidants in BAL [118], nor did we investigate the possible role of compartmentalization of these antioxidants. In addition, although carbonylated protein levels were higher in WTO_3 _mice than in KOO_3_, we did not assess levels of other oxidized molecular species, such as lipid peroxidation products, whose formation is known to be inhibited by SP-A [101,102,119]. If indeed, SP-A plays an antioxidant role in WT mice by scavenging reactive species under both normal and perturbed conditions as has been previously suggested [120], its absence in the KO mice may result in increased oxidative stress, even under normal conditions. The findings in the present study support this postulate. Moreover, the lack of SP-A may contribute to an added oxidative stress following O_3 _exposure via the reduction in PMN recruitment as shown in this and in a previous study [8]. Thus, based on both similarities and differences in protein levels among the groups under study, it is likely that different and overlapping mechanisms are operative.

## Conclusion

Using discovery proteomics and a mouse genetic model of a deficiency of an innate host defense molecule (SP-A) we have examined, for the first time using the 2D-DIGE approach, global changes in the BAL proteome of WT and KO mouse strains that occur in response to ozone exposure, an acute oxidative stress. By characterizing these protein expression changes with the broader, unbiased perspective of a "discovery" approach we were able to gain insight into a more complete understanding of pathophysiologic changes caused by ozone exposure. For example, the widespread decreases in RED proteins involved in redox balance suggest enhanced turnover of these proteins as a consequence of the oxidative stress resulting from ozone exposure, and the increases in PMM proteins involved in protein metabolism and modification are likely related to this increased turnover. The many changes (both increases and decreases) in proteins in the DEF group provide a possible basis for the increased susceptibility of some individuals to infection following an oxidative stress. Furthermore, the differences described in the response patterns of WT mice and SP-A KO mice provide support for a role of SP-A in innate immunity and redox balance under normal conditions (in the absence of an exogenous oxidative stress) as well as in the presence of an ozone-induced oxidative stress. Thus, based on the present findings, we submit that the sensitivity to oxidative stress in the four conditions we studied here is: KOO_3 _≥ KOFA ≈ WTO_3 _≥ WTFA. Moreover, the susceptibility of SP-A to oxidation shown by previous studies, together with its abundance in BAL fluid, make it ideally suited to play a role as a "sacrificial antioxidant," as has been described for albumin [104,105,109] and postulated for other proteins [107]. Further study is warranted to investigate the postulated mechanisms in greater detail.

## Abbreviations

2D-DIGE: two-dimensional difference gel electrophoresis; BAL: bronchoalveolar lavage; DEF: defense and immunity; KO: SP-A -/- (knockout) mice; KOFA: SP-A -/- mice exposed to filtered air; KOO_3_: SP-A -/- mice exposed to O_3_; MALDI-ToF/ToF: matrix-assisted laser desorption ionization-time of flight/time of flight; PCA: principal component analysis; PMM: protein metabolism and modification and chaperones; PMN: polymorphonuclear leukocytes; ppm: parts per million; RED: redox balance; SP-A: surfactant protein-A; WT: wild type mice; WTFA: wild type mice exposed to filtered air; WTO_3_: wild type mice exposed to O_3_

## Competing interests

The authors declare that they have no competing interests.

## Authors' contributions

RH exposed animals to ozone, collected samples, ran gels, did preliminary analysis, and assisted with the manuscript. TMU assisted RH, organized and analyzed data, and participated in the writing of the manuscript. WMF did MALDI-ToF/ToF analysis and assisted with evaluation of 2D-DIGE and mass spec data. JF assisted with study design and data interpretation and participated in manuscript preparation. DSP designed the study, interpreted data, and prepared the manuscript. All authors read and approved the final manuscript.

## Supplementary Material

Additional file 1 **MIAPE GE**. File containing Minimum Information About a Proteomics Experiment – Gel Electrophoresis in the format recommended by the Human Proteome Organization Proteomic Standards Initiative. Click here for file

Additional file 2 **MIAPE GI**. File containing Minimum Information About a Proteomics Experiment – Gel Informatics in the format recommended by the Human Proteome Organization Proteomic Standards Initiative. Click here for file

Additional file 3 **List of identified proteins**. Table contains a list of all proteins on the reference gel that have been identified by MALDI-ToF/ToF, their accession numbers, and the biological processes and molecular functions attributed to each by PANTHER. We have also assigned most of these to one of three functional groups: Defense and immunity (DEF); Redox balance (RED); Protein metabolism and modification and chaperones (PMM) and provided the reference from which that assignment was made (See reference list in main paper). Click here for file
