# Supplementary material for: The impact of surfactant protein-A on ozone-induced changes in the mouse bronchoalveolar lavage proteome
Source: Proteome Sci. 2009 Mar 26;7:12. doi: 10.1186/1477-5956-7-12 (PMC2666657; doi:10.1186/1477-5956-7-12)
Supplement: Additional file 2 — MIAPE GI. File containing Minimum Information About a Proteomics Experiment – Gel Informatics in the format recommended by the Human Proteome Organization Proteomic Standards Initiative. [file 1477-5956-7-12-S2.doc]

Additional File 2

MIAPE: Gel Informatics

*Version 0.4, 24^th^ April, 2007*

Reporting requirements for gel informatics data

1. General features

- Date stamp: 2006-05-16
- Responsible person or institutional role: Todd M. Umstead, Senior Research Support Associate, Penn State Center for Host defense, Inflammation, and Lung Disease (CHILD) Research, Department of Pediatrics, P.O. Box 850, Hershey, PA 17033
- Electrophoresis type: 2D-DIGE with PAGE electrophoresis
- Electrophoresis context: The Impact of Surfactant Protein-A on Ozone-Induced Changes in the Mouse Bronchoalveolar Lavage Proteome
- Image(s): Available upon request
- Image analysis software: Progenesis SameSpots v2.0 (Nonlinear Dynamics)
- Statistical analysis software: Progenesis SameSpots v2.0 (Nonlinear Dynamics), Excel (Microsoft)

1. Gel analysis design
   - Type: Directed
   - Replicates: n=4 per group
   - Groups: 4 groups (wild type filtered air, wild type ozone, SP-A knockout filtered air, SP-A knockout ozone)
   - Internal standard: Cy2 normalization pool of equal protein from all study samples run on each analytical gel
   - External standard: Cy3/Cy5 counterbalancing to eliminate dye-based artifacts
2. Image pre-processing
   - Type: Images obtained using the Typhoon 9410 Variable Mode Imager (GE Healthcare) in the GEL file format (.gel) and cropped using ImageQuant TL (GE)
   - Analytical (quantitative) gels: Laser voltages were optimized for each fluorophore prior to scanning to avoid signal saturation. Identical laser settings were then used to scan each gel
   - Preparative/picking gels: Fixed with methanol/acetic acid and post-stained with SyproRuby, scanned independently from analytical gels
   - All gels were scanned at 100μm resolution
   - See MIAPE Gel Electrophoresis supplement for more specific details of image collection
   - Software: ImageQuant TL (GE)
3. Image analysis pre-processing

- Input image(s): Images obtained using the Typhoon 9410 Variable Mode Imager (GE) in the GEL file format (.gel) and cropped using ImageQuant TL (GE)
- Type: See input images above
- Software***:*** ImageQuant TL (GE)

1. Data extraction process

- Input image(s): Images obtained using the Typhoon 9410 Variable Mode Imager (GE) in the GEL file format (.gel) and cropped using ImageQuant TL (GE) and are available upon request
- Image quality control: Image QC done using Progenesis SameSpots v2.0 (Nonlinear Dynamics) to check for proper file type, image manipulation prior to analysis, and image saturation
- Image alignment
  - Automatic gel alignment using Progenesis SameSpots v2.0 (Nonlinear Dynamics) to allow for more accurate spot matching
  - Gel alignment manually edited following automated alignment protocols
- Feature detection
  - Automatic spot detection using Progenesis SameSpots v2.0 (Nonlinear Dynamics)
  - Features were manually edited following automated spot detection protocols
- Matching
  - Algorithm: Progenesis SameSpots v2.0 (Nonlinear Dynamics)
  - Reference image used: 27550 Standard Cy2 aligned
  - Landmarks: Vectors were automatically and manually placed
  - Match editing: Automatic spot matching using Progenesis SameSpots v2.0 (Nonlinear Dynamics) followed by manual edited to confirm matches
  - One-hundred percent spot matching across all gels without missing values was set as a requirement for spot inclusion for data analysis
- Feature quantitation
  - Type: Normalized Volume
  - Quantitation: Progenesis SameSpots v2.0 (Nonlinear Dynamics)
  - Background subtraction: N/A
  - Normalization: Progenesis SameSpots v2.0 (Nonlinear Dynamics)

1. Data analysis

- Analysis intent: Features with ANOVA p<0.05
- Software: Progenesis SameSpots v2.0 (Nonlinear Dynamics), Excel (Microsoft)
- Type: ANOVA with false-discovery rates based on Progenesis assigned q-value, t-test, principal component analysis (PCA)
- Parameters: Not blinded
- Input data: Normalized volume (Cy3/Cy2 and Cy5/Cy2)

1. Data reporting

- List of image features: Excel file available upon request
- List of matches: Excel file available upon request
- Description of analysis results: Excel file available upon request
