# Supplementary material for: The impact of surfactant protein-A on ozone-induced changes in the mouse bronchoalveolar lavage proteome
Source: Proteome Sci. 2009 Mar 26;7:12. doi: 10.1186/1477-5956-7-12 (PMC2666657; doi:10.1186/1477-5956-7-12)
Supplement: Additional file 3 — List of identified proteins. Table contains a list of all proteins on the reference gel that have been identified by MALDI-ToF/ToF, their accession numbers, and the biological processes and molecular functions attributed to each by PANTHER. We have also assigned most of these to one of three functional groups: Defense and immunity (DEF); Redox balance (RED); Protein metabolism and modification and chaperones (PMM) and provided the reference from which that assignment was made (See reference list in main paper). [file 1477-5956-7-12-S3.doc]

**Additional File 3: List of identified proteins.**

| **Gel ID** | **Protein** | **Access. No** | **Molecular**  **Function** | **Biological**  **Process** | **Functional Group** | **Ref** |
| --- | --- | --- | --- | --- | --- | --- |
| 1 | 14-3-3 zeta | [Swiss-Prot:P63101] | OCH | ST, OPTL, CC | PMM | [55,56] |
| 2 | Adipsin (complement factor D) | [Swiss-Prot:P03953] | SP, COMP | PROT, CMI | DEF, PMM | [57-59] |
| 3 | Aldehyde dehydrogenase AHD-M1 | [Swiss-Prot:P47738] | DH | OCM | RED | [60] |
| 4 | Aldehyde dehydrogenase, dimeric NADP-preferring (EC 1.2.1.5) (ALDH class 3) (Dio | [Swiss-Prot:P47739] | DH | OCM | RED | [60] |
| 5 | Aldose reductase (EC 1.1.1.21) (AR) (Aldehyde reductase) | [Swiss-Prot:P45376] | RD | OM | RED | [61] |
| 6 | Alpha-1-antitrypsin 1-1 precursor (Serine protease inhibitor 1-1) | [Swiss-Prot:P07758] | PI | PROT | PMM,DEF | [62] |
| 7 | Alpha-1-antitrypsin 1-6 precursor (Serine protease inhibitor 1-6) (Alpha-1 protease inhibitor 6) | [Swiss-Prot:P81105] | PI | PROT | PMM,DEF | [62] |
| 8 | Annexin A1 (Annexin I) (Lipocortin I) (Calpactin II) (Chromobindin-9) (p35) (Pho | [Swiss-Prot:P10107] | TCP, ANX | FAM, CSM, ST | DEF | [63] |
| 9 | Annexin A2 (Annexin II) (Lipocortin II) (Calpactin I heavy chain) (Chromobindin) | [Swiss-Prot:P07356] | TCP, ANX | OPTL, DP, CSM |  |  |
| 10 | Annexin A3 | [Swiss-Prot:O35639] | TCP, ANX | LFSM |  |  |
| 11 | Annexin A4 | [Swiss-Prot:Q3UCL0] | TCP, ANX | LFSM |  |  |
| 12 | Annexin A5 | [Swiss-Prot:P48036] | TCP, ANX | LFSM | DEF | [63] |
| 13 | Antithrombin-III precursor (ATIII) | [Swiss-Prot:P32261] | PI | PROT | PMM, DEF | [64] |
| 14 | Apolipoprotein A-I | [Swiss-Prot:Q58EV2] | TP, ALP | LFAT | DEF, RED | [65,66] |
| 15 | Apolipoprotein A-IV | [Swiss-Prot:P06728] | TP, ALP | LFAT, BCGE | RED | [67] |
| 16 | Beta actin | [Swiss-Prot:P99041] | ARP | EE, TRAN, CK, CSM |  |  |
| 17 | Carbonyl reductase 2 | [Swiss-Prot:P08074] | DH, RD | OM | RED | [68] |
| 18 | Ceruloplasmin isoform | [Swiss-Prot:Q61147] | OT, OTCP, OXI | OHA | RED | [69] |
| 19 | Chain A, The Crystal Structure Of Novel Mammalian Lectin Ym1 Suggests A Saccharide Binding Site | [Swiss-Prot:O35744] | GL | OPM | DEF | [70,71] |
| 20 | Chia protein | [Swiss-Prot:Q91XA9] | GL | OPM | DEF | [70,71] |
| 21 | Chitinase-3-like protein 1 precursor (Cartilage glycoprotein 39) (GP-39) | [Swiss-Prot:Q61362] | GL | OPM | DEF | [70,71] |
| 22 | Coiled-coil domain containing 122 | [Swiss-Prot:Q8BVN0] | UN | UN |  |  |
| 23 | Complement component 3 | [Swiss-Prot:Q80XP1] | COMP | CMI | DEF | [72] |
| 24 | Complement component C5 | [Swiss-Prot:P06684] | COMP | CMI | DEF | [73,74] |
| 25 | Contrapsin | [Swiss-Prot:P07759] | PI | PROT | PMM | [75,76] |
| 26 | Creatine kinase M-type (EC 2.7.3.2) (Creatine kinase M chain) (M-CK) | [Swiss-Prot:P07310] | OK | MC |  |  |
| 27 | Cytosolic malate dehydrogenase | [Swiss-Prot:P14152] | DH | TAP | RED | [77] |
| 28 | Esterase 1 | [Swiss-Prot:P23953] | ES | DTX | DEF | [78] |
| 29 | Gamma-actin | [Swiss-Prot:P63260] | ARP | EE, TRAN, CK, CSM |  |  |
| 30 | Gelsolin precursor (Actin-depolymerizing factor) (ADF) (Brevin) | [Swiss-Prot:P13020] | NMAB | CSM | RED |  |
| 31 | Glutathione S-transferase A4 (GST A4-4) (GSTA4-4) | [Swiss-Prot:P24472] | OTF | DTX | RED, DEF | [79] |
| 32 | Glutathione S-transferase omega 1 | [Swiss-Prot:O09131] | OTF | DTX, AFRR | RED,DEF | [80] |
| 33 | Glyceraldehyde-3-phosphate dehydrogenase (EC 1.2.1.12) (GAPDH) | [Swiss-Prot:P16858] | UN | UN | RED | [81] |
| 34 | Haptoglobin | [Swiss-Prot:Q60574] | SP | PROT, SR, BCGE | DEF, PMM | [82-84] |
| 35 | Heat shock protein 1, alpha (HSP 60) | [Swiss-Prot:Q80Y52] | PI | PF, SR | PMM,DEF | [85,86] |
| 36 | Heat shock protein 8 (HSP 70) | [Swiss-Prot:P63017] | HSP | PF, PCXA, SR | RED, PMM,DEF | [52,87,88] |
| 37 | Hemopexin | [Swiss-Prot:Q8K1U6] | OTCP | VCT, OCP, TRAN | RED | [89] |
| 38 | Isocitrate dehydrogenase [NADP] cytoplasmic (EC 1.1.1.42) (Cytosolic NADP-isocitr | [Swiss-Prot:O88844] | DH | TAP | RED | [90,91] |
| 39 | Kininogen 1 | [Swiss-Prot:O08677] | CP1 | BC, RVCD | DEF,PMM | [84,92] |
| 40 | Kpnb1 protein | [Swiss-Prot:Q99KM9] | TCP | NT, PTL, TRAN |  |  |
| 41 | Lactate dehydrogenase 2, B chain | [Swiss-Prot:P16125] | DH | OCM | RED,DEF | [93] |
| 42 | Murinoglobulin-1 precursor (MuG1) | [Swiss-Prot:P28665] | OCY, SPI | PROT, LMS, ID | PMM, DEF | [94] |
| 43 | Oxysterol-binding protein-related protein 1 (OSBP-related protein 1) (ORP-1) | [Swiss-Prot:Q91XL9] | TCP, OMFP | CM |  |  |
| 44 | Peroxiredoxin 6 (Antioxidant protein 2) (AOP2) | [Swiss-Prot:O08709] | PER | AFRR | RED,DEF | [68] |
| 45 | Plasminogen | [Swiss-Prot:P20918] | PH, OPI, OEI, SP, CRP, ANX | LFAT, PROT, TRAN, BC, APP, AGG, BCGE, CPD | DEF,PMM | [95] |
| 46 | Pregnancy zone protein | [GenBank:gi|110347469] | OCY, PI | PROT, LMS, ID | PMM,DEF | [96] |
| 47 | Protein disulfide-isomerase A3 precursor (EC 5.3.4.1) (Disulfide isomerase ER-60 | [Swiss-Prot:P27773] | OI | PDIR | RED, PMM | [97] |
| 48 | Prothrombin precursor (EC 3.4.21.5) (Coagulation factor II) | [Swiss-Prot:P19221] | SP | PROT, BC | DEF,PMM |  |
| 49 | Pulmonary surfactant-associated protein A precursor (SP-A) (PSP-A) (PSAP) | [Swiss-Prot:P35242] | ODIP, SURF | MMI, BCGE | DEF | [30] |
| 50 | beta-actin (putative, AA 27-375) | [Swiss-Prot:P60710] | ARP | EE, TRAN, CK, CSM |  |  |
| 51 | Retinal dehydrogenase 1 (EC 1.2.1.36) (RalDH1) (RALDH 1) (Aldehyde dehydrogenase | [Swiss-Prot:P24549] | DH, RD | OCM | RED | [98] |
| 52 | Rho GDP dissociation inhibitor (GDI) alpha | [Swiss-Prot:Q99PT1] | OSM, OGPM | ISC |  |  |
| 53 | SEC14-like 3 | [Swiss-Prot:Q5SQ27] | OT, TCP | CM, OT |  |  |
| 54 | Selenium binding protein 1 | [Swiss-Prot:P17563] | ODIP, OMFP | ID | DEF,RED | [68] |
| 55 | Selenium binding protein 2 | [Swiss-Prot:Q63836] | ODIP, OMFP | ID | DEF | [99] |
| 56 | Serine (or cysteine) proteinase inhibitor, clade A, member 1e | [Swiss-Prot:Q00898] | PI | PROT | PMM |  |
| 57 | Similar to Ferritin light chain 1 (Ferritin L subunit 1) | [GenBank:gi|94396246] | STOR | CATT, OHA | RED,DEF | [100] |
| 58 | Similar to Glutathione S-transferase Ya chain (GST class-alpha) (Ya1) | [GenBank:gi|94387421] | OTF | DTX | RED,DEF |  |
| 59 | Similar to Keratin, type I cytoskeletal 10 (Cytokeratin-10) (CK-10) (Keratin-10) (K10 | [GenBank:gi|94391959] | IF, STP | CSM |  |  |
| 60 | Similar to RNP particle component | [GenBank:rf|XP_359003.1] | UN | UN |  |  |
| 61 | Similar to T21B10.2b | [GenBank:gi|94374068] | LY | OCM |  |  |
| 62 | Toll-like receptor 13 precursor | [Swiss-Prot:Q6R5N8] | RT, EM | CCMSP, DP |  |  |
| 63 | Transferrin | [Swiss-Prot:Q921I1] | TCP, SP, MISC | TRAN, MISC | PMM,RED |  |
| 64 | Transitional endoplasmic reticulum ATPase (TER ATPase) (15S Mg(2+)-ATPase p97 | [Swiss-Prot:Q01853] | UN | PMM, EE, PTL, TRAN | PMM |  |
| 65 | Tyrosine 3-monooxygenase/tryptophan 5-monooxygenase activation protein, Epsilon polypeptide | [Swiss-Prot:Q8BPH1] | OCH | ST, OPTL, CC | PMM |  |
| 66 | Vimentin | [Swiss-Prot:P20152] | IF, SP | DP, CSM |  |  |

**Additional File 3.**  List of identified proteins. Gel numbers, protein names, accession numbers, and molecular function and biological process designations from the PANTHER database are listed. The functional group column places proteins into categories we defined as broadly related to redox regulation (RED), those involved in defense and immunity function (DEF), and those involved in protein modification and metabolism (PMM). References are given to support the assignment of some proteins to each of the functional groups. Abbreviations for the biological processes and molecular functions assigned by PANTHER are defined below:

| **Molecular Function** | | |  |
| --- | --- | --- | --- |
| ALP | Apolipoprotein | |  |
| ANX | Annexin | |  |
| ARP | Actin and actin-related protein | |  |
| COMP | Complement component | |  |
| CRP | Calmodulin related protein | |  |
| DH | Dehydrogenase | |  |
| EM | Extracellular matrix | |  |
| ES | Esterase | |  |
| GL | Glycosidase | |  |
| HSP | Hsp family chaperone | |  |
| IF | Intermediate filament | |  |
| IGRF | Immunoglobulin receptor family member | |  |
| LY | Lyase | |  |
| MISC | Miscellaneous | |  |
| NMAB | Non-motor actin binding protein | |  |
| OCH | Other chaperones | |  |
| OCY | Other cytokine | |  |
| ODIP | Other defense and immunity protein | |  |
| OEI | Other enzyme inhibitor | |  |
| OI | Other isomerase | |  |
| OK | Other kinase | |  |
| OMFP | Other miscellaneous function protein | |  |
| OPI | Other protease inhibitor | |  |
| OSCB | Other select calcium binding protein | |  |
| OGPM | Other G-protein modulator | |  |
| OSM | Other signaling molecules | |  |
| OT | Other transporter | |  |
| OTCP | Other transfer/carrier protein | |  |
| OTF | Other transferase | |  |
| OXI | Oxidase | |  |
| PER | Peroxidase | |  |
| PH | Peptide hormone | |  |
| PI | Protease inhibitor | |  |
| RD | Reductase | |  |
| RT | Receptor | |  |
| SP | Serine protease | |  |
| STOR | Storage protein | |  |
| STP | Structural protein | |  |
| SURF | Surfactant | |  |
| TCP | Transfer/carrier protein | |  |
| TP | Transporter | |  |
| UN | Molecular function unclassified | |  |
| **Biological Process** | | | |
| AFRR | | Antioxidation and free radical removal | |
| AGG | | Angiogenesis | |
| APP | | Apoptosis | |
| BC | | Blood clotting | |
| BCGE | | Blood circulation and gas exchange | |
| CATT | | Cation transport | |
| CC | | Cell cycle | |
| CCMSP | | Cytokine/chemokine mediated signaling pathways | |
| CK | | Cytokinesis | |
| CM | | Cholesterol metabolism | |
| CMI | | Complement-mediated immunity | |
| CPD | | Cell proliferation and differentiation | |
| CSM | | Cell structure and motility | |
| DP | | Development process | |
| DTX | | Detoxification | |
| EE | | Exocytosis and endocytosis | |
| FAM | | Fatty acid metabolism | |
| ID | | Immunity and defense | |
| ISC | | Intracellular signaling cascade | |
| LFAT | | Lipid and fatty acid transport | |
| LFSM | | Lipid, fatty acid and steroid metabolism | |
| LMS | | Ligand-mediated signaling | |
| MC | | Muscle contraction | |
| MISC | | Miscellaneous | |
| MMI | | Macrophage-mediated immunity | |
| NT | | Nuclear transport | |
| OCM | | Other carbon metabolism | |
| OHA | | Other homeostasis activities | |
| OM | | Other metabolism | |
| OPM | | Other polysaccharide metabolism | |
| OPTL | | Other protein targeting and localization | |
| OT | | Other transport | |
| PCXA | | Protein complex assembly | |
| PDIR | | Protein disulphide-isomerase reaction | |
| PF | | Protein folding | |
| PMM | | Protein metabolism and modification | |
| PROT | | Proteolysis | |
| PTL | | Protein targeting and localization | |
| RVCD | | Regulation of vasoconstriction | |
| SR | | Stress response | |
| ST | | Signal transduction | |
| TAP | | Tricarboxylic acid pathway | |
| TRAN | | Transport | |
| UN | | Biological process unclassified | |
| VCT | | Vitamin/cofactor transport | |
